# Pregnancy with Complete Heart Block-An Emergency Cesarean Section with Temporary Pacemaker: A Case Report

**DOI:** 10.31729/jnma.5172

**Published:** 2020-08-31

**Authors:** Anamika Das, Pritha Basnet, Ramesh Shrestha, Abha Hada, Bidhur Bhandari

**Affiliations:** 1 Department of Obstetrics and Gynecology, B.P. Koirala Institute of Health Sciences, Dharan, Nepal

**Keywords:** *heart block*, *pacemaker*, *pregnancy*

## Abstract

Management of a pregnant woman with complete heart block presenting during pregnancy and without pacing remains debatable. To bear up against any hemodynamic variations in peripartum period, temporary pacemakers have been advocated by some authors. Herein, we report a case of successful management of a 24 year old, pregnant woman with CHB who had an uneventful emergency caesarean delivery under spinal anesthesia after temporary pacing. She was an unbooked patient detected with CHB first time during active stage of labour. She delivered a healthy male baby and was discharged from the hospital in a stable and satisfactory condition on seventh postoperative day.

## INTRODUCTION

Complete heart block (CHB) is a relatively rare and a potentially serious issue in pregnancy.^1^ This condition poses a significant challenge to the treating physician. Obstetric outcome in women who have undergone permanent pacemaker implanted has been reported, but there is a paucity of literature on the management of complete heart block detected for the first time during labour in pregnancy.^2^ We herein present a case of pregnancy with CHB detected for the first time during active stage of labour that has been managed by temporary pacemaker insertion with the co-ordinated action of cardiologist and obstetrician.

## CASE REPORT

**Figure 1.**
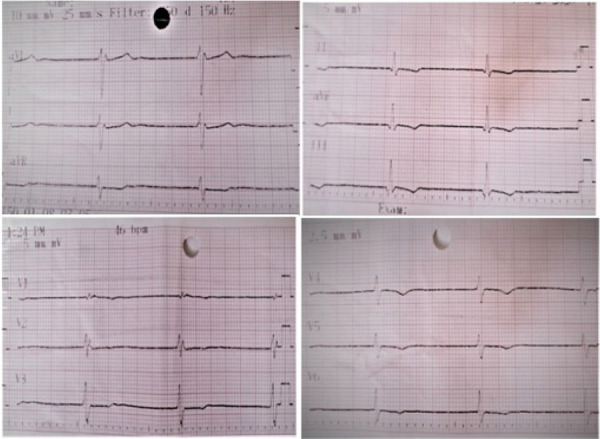
ECG showing complete heart block with a ventricular rate of about 46/min.

A 24 year old, unbooked, immunized G4P3L2 IUD1 at 36weeks and 4 days period of gestation, was referred from a private hospital in view of prolonged labour with maternal bradycardia. She presented with complain of dizziness since last two days in early active stage of labour. She had irregular antenatal check up and her antenatal check up was uneventful. In all her previous deliveries, she had no card iorespiratory complain. No history of any medical illnesses, addiction or drug intake such as beta-blocker, calcium-channel blocker or digitalis. There is no significant family history. On examination at Obstetric emergency, her general condition was fair, was normotensive, with maternal bradycardia (PR = 42 bpm). Cardivascular system examination showed bradycardia and respiratory system was unremarkable. On abdominal examination, there was a single live fetus in cephalic presentation and uterine height corresponded to 36 weeks of gestation with a fetal heart rate 140 beats/minute. There was regular uterine contractions. On per vaginum examination, os was 4 centimeters dilated, cervix was 50% effaced, vertex was at -2 station , membranes was ruptured and liquor was clear. Cardiology consulation was done in view of persistent bradycardia. Electrocardiogram (ECG) was done immediately which showed sinus bradycardia, Atrio-ventricular (AV) dissociation, and ventricular rate 40% suggestive of complete heart block (CHB) (Fig. 1).

All her routine investigation along with cardiac enzymes, and thyroid function test was within normal limit. Echocardiagraphy was normal. Patient was shifted to cath lab and temporary pacemaker insertion (TPI) was done via Right femoral vein route (Local Anesthesia) with fetal shielding and heart rate was fixed at 80bpm. Emergency cesarean section was done in view of arrest of descent and dilatation under spinal anesthesia under high risk consent. Intraoperative haemodynamics remained stable and surgery proceeded uneventfully. She gave birth to a healthy male child. The neonate did not have any rhythm disturbances. Patient was transferred to Maternal Intensive Care Unit (MICU) for observation. Postoperatively, continuous monitoring was done with pulse oximeter and invasive blood pressure. She was started on intravenous antibiotics (third generation cephalosporins and metronidazole) and thromboprophlyaxis with low molecular weight heparin. Heart rate remained steady at 46-58bpm. Rest of the post operative period was uneventful. TPI was removed under aseptic precaution on 5^th^ post operative day. She was discharged from the hospital in a stable and satisfactory condition on seventh postoperative day. Later patient followed up in Gynae OPD after 6weeks in a stable condition following which she was referred to cardiologist for permanent pacemaker implantation.

## DISCUSSION

CHB detected for the first time during pregnancy and delivery is a rare disease with an incidence of 1 in 20,000 live births.^3^ The etiology of complete heart block is not completely understood yet however it's considered to be mostly congenital in origin. Most often these patients are asymptomatic; however the symptoms can also occur later in life. This is due to a variable degree of heart block. Fetomaternal outcome is favorable in asymptomatic cases and in uncomplicated bradyarrhythmias without significant underlying heart disease.^4^-^5^ Rarely, preterm birth and intrauterine growth restriction has been observed. Most of the symptoms are related to the slow heart rate resulting in hydrops foetalis, heart failure of the neonate or exercise intolerance of the child. These symptoms are present in patients with resting heart rate of 50 beats/min or less. Mortality from congenital complete heart block is highest in the neonatal period, is much lower in childhood and adolescence and increases gradually later in life. Asymptomatic pregnant patients without pacemakers may present with sudden cardiac death or heart failure during pregnancy, or may become symptomatic during labour due to valsalva induced bradycardia.

Patients with complete heart block presenting for the first time in pregnancy is a therapeutic challenge to the physician. Symptomatic patients in pregnancy should be managed with the use of cardiac pacemaker, which should be implanted whenever heart block is diagnosed. Pacemaker is needed to maintain cardiac function. However, prophylactic placement of a pacemaker is not indicated in asymptomatic patients.^6,7^ In women without a permanent pacemaker, temporary pacemakers have been routinely inserted for labour and birth probably to withstand any hemodynamic variations.^8^ However, the need of temporary pacemaker during labour and it's accurate timing and rate setting of pacemaker has not been objectively evaluated so far. Permanent pacemakers can be implanted at any time in pregnancy whereas short-term temporary pacemaker can be applied during delivery. Overall maternal and neonatal outcome is good in such patients.

In our case CHB is identified at near term pregnancy with quite a low heart rate (42 beats/min) and with history of dizziness. Emergency caesarean section was carried out under temporary pacing coverage with a later plan of a permanent pacemaker implantation resulting in excellent symptom free status. To our knowledge, this is the first such case report from B P Koirala Institute of Health Sciences (BPKIHS) where close co-operation between cardiologist and obstetrician of our hospital has resulted in a happy ending by successful implantation of pacemaker in a pregnant woman. Women with asymptomatic CHB presenting at the time of labour poses a challenge to the treating Obstetrician. As suggested by our case, caesarean delivery might be safely contemplated with temporary pacing in symptomatic women with CHB. However, close monitoring with multidisciplinary approach and follow-up of cardiac function is needed in these pregnant women during labour and perioperative period.
